# Vulval Intestinal/Enteric Heterotopia in a Patient with Crohn's Disease

**DOI:** 10.1155/2020/6203826

**Published:** 2020-03-19

**Authors:** Jean-Christophe Noël, Cristina Rotea, Laurine Verset, Xavier Catteau

**Affiliations:** ^1^Department of Pathology, Erasme University Hospital/Curepath, ULB, Brussels, Belgium; ^2^Department of Gynecology, Clinique du Lothier/ULB Erasme, Brussels, Belgium; ^3^Department of Pathology, Bordet Institute, ULB, Brussels, Belgium

## Abstract

Intestinal/enteric heterotopia of the vulva is an extremely rare disease with only 3 cases described in the literature. We report here an unusual case of this disease occurring in a 26-year-old patient in a context of Crohn's disease. To the best of our knowledge, such type of association has not been previously described. The potential origins of these lesions including metaplastic transformation, dysontogenetic changes, or epithelial colonic displacement/implantation are discussed.

## 1. Introduction

Vulval glandular lesions with gastrointestinal features are extremely rare and include mainly mucinous metaplasia, intestinal/enteric heterotopia, villous adenoma, and cloacogenic neoplasms with enteric features [1–11]. From these, vulval intestinal/enteric heterotopia are the more infrequent with only 3 cases described in the literature [1, 2]. To the best of our knowledge, we report here the first case of this entity occurring in the context of chronic vulvar inflammation associated with Crohn's disease [11, 12].

## 2. Case Presentation

**Figure 1:**
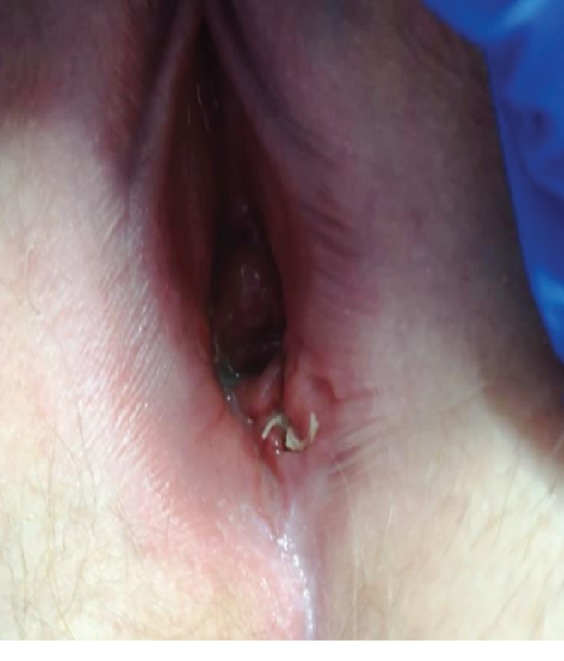
Ulcerated lesions located in the lower part of left labia minora in a context of atrophy with a pale pink hue (post biopsy).

A 26-year-old presented to our gynecologic consultation for a painful ulcerated lesion appeared 3 months earlier. It was located in the lower part of the left labia minora with extension to the posterior “fourchette” and to the introit. This lesion occurred in a context of atrophic-like vulva with a pale pink hue (Figure 1).

**Figure 2:**
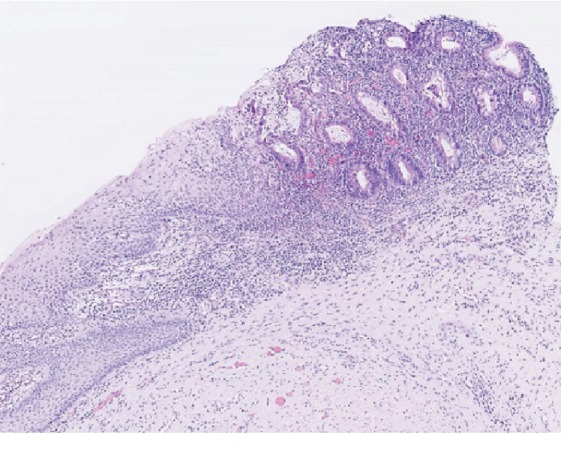
Histological features of vulval intestinal/enteric heterotopia view. Sharp transition of the normal squamous epithelium into enteric-type in a context of chronic inflammation.

The patient has a past history of colonic Crohn's disease diagnosed 14 years before with secondary multiple episodes of perianal fistula or hidradenitis suppurativa. The patient was treated with diverse immunosuppressive treatments including azathioprine, methylprednisolone, and adalimumab or surgery for the perianal fistula. The last episode having occurred a year ago but the patient is currently asymptomatic from a digestive point of view. A year earlier, the patient had also presented two episodes of vulvar herpetic infections successfully treated with valacyclovir. The viral culture was negative to date. A biopsy of the lesion was taken and revealed an abrupt transition of the normal squamous epithelium into mucinous enteric-type columnar glandular epithelium in a context of chronic inflammation (Figure 2). Noncaseating granuloma, classically considered the more specific histological criteria for vulvar Crohn's disease, was not present here [12, 13]. In addition, there were no morphological arguments for a lichen sclerosus or a herpetic infection.

**Figure 3:**
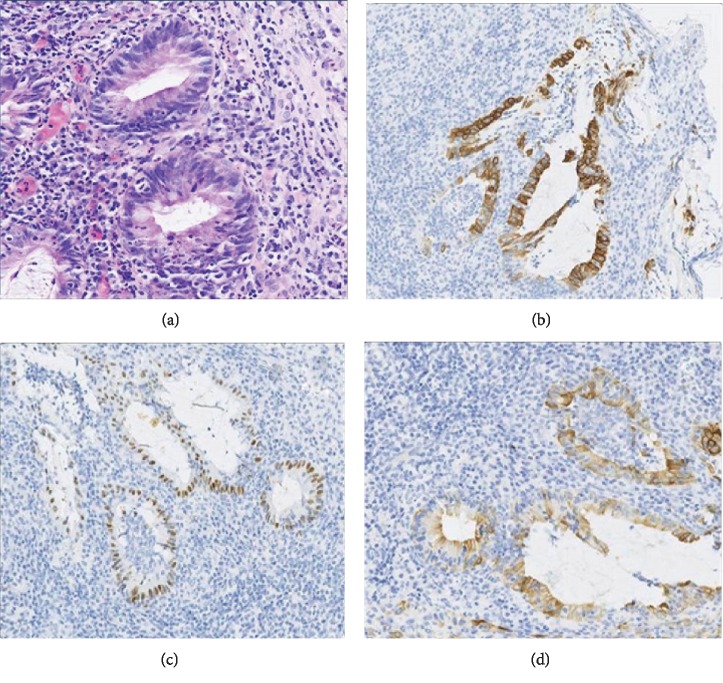
Intestinal/enteric vulval heterotopia. At high power view, some glands were lined by a mucinous columnar epithelium with nuclear stratification, mild atypia, and rare mitosis compatible with either inflammatory or regenerative atypia even if a possible associated low-grade dysplasia cannot be excluded (a). Note the strong positive staining for the CK 20 (b) and CDX2 (c). The CK 7 staining was less marked (d).

At high power view, some of the glands were lined by a mucinous columnar epithelium with focally nuclear pseudostratification, mild atypia, and rare mitosis suggestive of a possible either inflammatory or regenerative changes even if a possible associated low-grade dysplasia cannot be excluded. These glands were strongly positive for CK 20, CDX2, and CEA. The CK 7 and p53 were also focally positive. Staining for ER, PR, GCDFP-15, GATA 3, and PAX-8 was negative (Figure 3).

As the potential risk of malignant transformation of this entity is unknown, a close follow-up of the patient has been advised, but no recurrence has been observed to date.

## 3. Discussion

Vulval glandular lesions with gastrointestinal features of the vulva are extremely rare and include mucinous metaplasia, vulval enteric/intestinal heterotopia, tubulovillous adenomas, and adenocarcinoma with intestinal features [1–11]. From these, enteric/intestinal heterotopia are the more rare with only 3 previously described [1, 2]. This entity is histologically characterized, as in our case, by a direct transition of the normal squamous epithelium into intestinal-type glands which are also present in the chorion, generally in association with chronic inflammation. Intestinal metaplasia or heterotopia is different from mucinous metaplasia. The latter is characterized by the replacement of squamous cells by columnar or goblet mucinous cells, mostly in the superficial layers of squamous epithelium generally as a result of an inflammatory condition (lichen planus, sclerosus, Zoon's vulvitis,…) or more rarely in a context of vulvar intraepithelial neoplasia [3, 14–16].

The glands in intestinal metaplasia/heterotopia showed a variable positivity for CK 7, CK 20, and CDX2 but are typically negative for ER and PR as well as breast-associated markers (Mammoglobin, GCDFP-15, and GATA 3) [1].

The histogenesis of intestinal-type vulval lesions (as their vaginal counterpart) remains controversial [1, 17].

The first potential origin is a metaplastic/derivation origin with enteric metaplasia from glandular adnexal structures, periurethral glands, Bartholin glands, or Müllerian adenosis [1, 11, 17–20].

Dysontogenic hypothesis from cloacal remnants and/or displacement of multipotent embryonic stem cells has also been proposed to be the origin of enteric metaplasia [1, 13]. Moreover, if the so-called “cloacogenic” adenocarcinoma has been described, the presence of benign cloacogenic remnants remains controversial [8–10].

In the context, given to the prior history of Crohn's disease in our patient with multiple episodes of fistula and surgical interventions, we postulated that displacement and “implantation” of rectal mucosa to vulvar epithelium during repair or laceration could constitute another attractive possibility to explain this entity. Indeed, this hypothesis has been evocated to explain some benign intestinal glandular lesions in the vagina but will however have to be confirmed in the future by the description of new similar cases [21].

Due to the rarity of cases, the potential malignant behavior of vulval enteric/intestinal metaplasia is unknown. In the present case, in some glands, nuclear stratification, mild atypia, and rare mitosis were present and suggestive of inflammation-associated regenerative changes even if a possible associated low-grade dysplasia cannot be excluded formally. All these elements should warn us to be cautious, especially since fistula-associated anal carcinoma in patients with Crohn's disease is increasingly reported [22, 23]. Therefore, these patients should be kept under regular follow-up.
